# The Genetic Landscape of Systemic Rheumatic Diseases: A Comprehensive Multigene-Panel Study Identifying Key Gene Polymorphisms

**DOI:** 10.3390/ph17040438

**Published:** 2024-03-28

**Authors:** Elena Rita Simula, Seyedesomaye Jasemi, Davide Cossu, Pietro Carmelo Manca, Daria Sanna, Fabio Scarpa, Gianfranco Meloni, Roberto Cusano, Leonardo Antonio Sechi

**Affiliations:** 1 Dipartimento di Scienze Biomediche, Università di Sassari, 07100 Sassari, Italy; simulaelena@gmail.com (E.R.S.); s.jasemi@studenti.uniss.it (S.J.); dcossu@uniss.it (D.C.); darsanna@uniss.it (D.S.); fscarpa@uniss.it (F.S.); 2 S.C. Servizio Immunotrasfusionale, Azienda Ospedaliero-Universitaria di Sassari, 07100 Sassari, Italy; pietro.manca@aousassari.it; 3 Dipartimento di Medicina, Chirurgia e Farmacia, Università di Sassari, 07100 Sassari, Italy; gfmeloni@uniss.it; 4 Centro di Ricerca, Sviluppo, Studi Superiori in Sardegna (CRS4), Pula, 09100 Cagliari, Italy; robycuso@crs4.it; 5 Struttura Complessa di Microbiologia e Virologia, Azienda Ospedaliera Universitaria, 07100 Sassari, Italy

**Keywords:** systemic rheumatic diseases, polymorphism, autoimmunity

## Abstract

Systemic rheumatic diseases, including conditions such as rheumatoid arthritis, Sjögren’s syndrome, systemic sclerosis, and systemic lupus erythematosus, represent a complex array of autoimmune disorders characterized by chronic inflammation and diverse clinical manifestations. This study focuses on unraveling the genetic underpinnings of these diseases by examining polymorphisms in key genes related to their pathology. Utilizing a comprehensive genetic analysis, we have documented the involvement of these genetic variations in the pathogenesis of rheumatic diseases. Our study has identified several key polymorphisms with notable implications in rheumatic diseases. Polymorphism at chr11_112020916 within the *IL-18* gene was prevalent across various conditions with a potential protective effect. Concurrently, the same *IL18R1* gene polymorphism located at chr2_103010912, coding for the IL-18 receptor, was observed in most rheumatic conditions, reinforcing its potential protective role. Additionally, a further polymorphism in IL18R1 at chr2_103013408 seems to have a protective influence against the rheumatic diseases under investigation. In the context of emerging genes involved in rheumatic diseases, like *PARK2*, a significant polymorphism at chr6_161990516 was consistently identified across different conditions, exhibiting protective characteristics in these pathological contexts. The findings underscore the complexity of the genetic landscape in rheumatic autoimmune disorders and pave the way for a deeper understanding of their etiology and the possible development of more targeted and effective therapeutic strategies.

## 1. Introduction

Systemic rheumatic diseases (SRDs) are chronic, inflammatory autoimmune disorders. Among them, rheumatoid arthritis (RA), systemic lupus erythematosus (SLE), systemic sclerosis (SSc), and Sjögren’s syndrome (SS) will be discussed in the present study. Sex-related disparities are markedly evident in SRDs, exhibiting a female predominance over male cases. Specifically, in RA the ratio is 3:1 [1], in SLE it is 7:1 [2], in SSc the ratio stands at 3:1 [3], and in SS [4] it is particularly striking at 9:1 [5].

RA is predominantly characterized by persistent inflammation of the synovial membrane, resulting in the degradation of joint structures. In contrast, SSc is distinguished by the development of fibrosis in the dermal layers and internal organs [6,7]. SS syndrome is principally characterized by dysfunction of the exocrine glands, leading predominantly to symptoms of xerophthalmia and xerostomia [8]. SLE is remarkable for its widespread systemic involvement and heterogeneous clinical manifestations, encompassing dermatological, renal, neurological, and hematological abnormalities [9]. At the core of these disorders lies the disruption of immune system regulation, leading to the generation of autoantibodies and persistent inflammation. The interplay of genetic susceptibility, environmental factors, biological pathogens [10,11,12], and hormonal influences plays a vital role in the initiation and progression of these diseases. In this scenario, polymorphisms represent a critical area of investigation due to their capacity to alter gene functionality or expression [13].

### 1.1. Role of Cytokines and Their Receptors in Systemic Rheumatic Disease

In SRDs, cytokines, and their receptors are pivotal in shaping the disease panorama. The pathological state observed in affected organs and tissues is largely influenced by the equilibrium between proinflammatory and anti-inflammatory cytokines that, in conditions such as SRDs, exert both protective and pathological roles, underscoring their dual nature in disease modulation. Proinflammatory cytokines, including TNF-α, IL-1, IL-6, and IL-17, are central drivers of the inflammatory process’s characteristic of SRDs. They play a crucial role in promoting synovial inflammation, facilitating the production of autoantibodies, and contributing to tissue damage [14,15]. On the other hand, anti-inflammatory cytokines such as IL-10 and TGF-β play a pivotal role in mitigating inflammatory responses and maintaining immune homeostasis. However, when the regulation of cytokines is disrupted, this balance can be altered, resulting in the advancement of the disease. Additionally, polymorphisms in cytokine receptors have been linked to differences in disease susceptibility and treatment response [13]. IL-12A, a subunit of interleukin-12, is integral in the process of driving Th1 cell differentiation [16]. Its influence extends to shaping immune responses toward a more inflammatory phenotype, which is often seen in autoimmune pathologies [17]. The interferon-gamma gene, *IFNG*, encodes for a pivotal proinflammatory cytokine (IFN-γ) that plays a broad role in immunoregulation. The functioning of IFN-γ is mediated through its receptors, IFNGR1 and IFNGR2, and when the signaling pathways are dysregulated, it can significantly contribute to the pathogenesis of autoimmune diseases such as SLE and RA [18]. IL-18 functions as an inducer of IFN-γ production and plays a pivotal role in promoting Th1 cell responses. By stimulating the production of IFN-γ, IL-18 contributes to the development and maintenance of Th1-dominant immune responses. These responses are characterized by increased production of proinflammatory cytokines, which can be beneficial in fighting infections but may also lead to pathological inflammation when dysregulated [19]. An effective and no excessive immune response is ensured by the presence of a balance between cytokines and their respective signaling cascades. Indeed, an imbalance in cytokine regulation can lead to a state of chronic inflammation and tissue damage.

### 1.2. Role of Key Genes in Autoimmune Rheumatic Diseases

Pivotal elements of complex intracellular signaling cascade that govern the immune response, such as TRAF6, MAPT, IRF5, NR1I2, and PTPN22, play critical roles in the dysregulation observed in SRDs [20,21]. Dysregulation of TRAF6, as may be observed in rheumatic diseases, can lead to aberrant activation of the NF-κB pathway. This inappropriate activation can result in the increased production of pro-inflammatory cytokines, which are key mediators in the development and maintenance of inflammation [22]. Genetic variants of *PTPN22* have been implicated in altered immune regulation and are associated with an increased risk of developing autoimmune diseases [23]. The interplay of costimulatory signals is integral to the immune system’s balance. Auxiliary factors such as the lymphocyte activation gene-3 (*LAG3*) serve as critical components in the pathogenesis of SRDs [24]. Several key genes, which might not be directly classified within the typical immune regulatory pathways, play substantial roles in the pathogenesis. Among these, the angiotensin-converting enzyme, specifically *ACE* and *ACE2*, traditionally associated with blood pressure regulation through the renin–angiotensin system, have been implicated in modulating immune responses, especially under conditions of stress and inflammation [25]. Vitamin D receptor, encoded by the *VDR* gene, exerts profound effects on immune cells, including promoting the development of regulatory T cells and dampening inflammatory responses [26]. The human solute carrier family 11 member 1 protein, encoded by *SLC11A1*, affects macrophage activation and can influence the immune response to pathogens, which can be a triggering factor for autoimmune reactions [27]. Recently, interesting aspects of the *PARK2* gene, traditionally associated with Parkinson’s disease, have been documented in the context of SRDs. The altered function of PARK2 may affect the balance between pro-inflammatory and anti-inflammatory signaling pathways, thereby influencing disease onset and progression [28].

Our investigation delineates the contribution of specific genetic polymorphisms to SRDs, emphasizing the involvement of immune system-related genes. We endeavor to generate critical insights into their roles within the not fully elucidated pathological pathways, thereby clarifying their protective or susceptibility-conferring mechanisms in the context of rheumatic disorders.

## 2. Results

Our study involved the enrollment of 100 HCs and 88 patients with SRDs, further classified as 26 RA patients, 21 SS individuals, 29 patients with SSc, and 12 subjects with SLE. We documented the existence of multiple polymorphisms in our analysis conducted on the entire cohort of patients with systemic rheumatic diseases (Table 1 and Table 2). Specifically, we observed a statistically significant difference between patients and healthy controls in the context of several genes, including *ACE* (chr17_61560763, chr17_61562063, and chr17_61564533), *PARK2* (chr6_161781225, chr6_161990516, and chr6_162394341,), *ACE2* (chrX_15590120), *IL-18* (chr11_112020916), *IL-18R1* (chr2_103010912), *IL-6* (chr7_22767433), *IL-7R* (chr5_35857235 and chr5_35861159), *IRF5* (chr7_128586057), *TMPRSS2* (chr21_42866107), *TRAF6* (chr11_36518824), *IFNGR1* (chr6_137519780) and *IL-12RB2* (chr1_67852335). Based on the analysis conducted, it appears that all the aforementioned polymorphisms have a protective effect in the context of the SRDs, although there are four exceptions. We observed a potential causative effect with Pr(>|t|) values of 0.013 and 0.02, respectively, for the presence of the polymorphism in the gene *IL-18R1* (chr5_103013408) and *IL-7R* (chr_35871190). Concerning *IL-12A* (chr3_159713467) and *MAPT* (chr17_44101591) polymorphisms, they did not show statistical significance between the two study populations, but they are included in the polymorphisms that could potentially cause disease. Conversely, genes such as *LAG3* (chr12_6883722), *ACE2* (chrX_15589725 and chrX_15613148), *IFNG* (chr12_68551941), *IL-17A* (chr6_52053771), and the polymorphism in the *IL18R1* gene (chr16_27353479) exhibit a protective trend.

Through the analysis of the RA population, we identified the presence of the highest number of polymorphisms compared to other diseases (Table 3 and Table 4). Among these, some exhibited a protective effect, while others showed a causative effect. In the overview of genes involved with a protective effect that demonstrated a statistically significant difference between RA patients and healthy controls, we found *ACE* (chr17_61566031), *PARK2* (chr6_161990516), *ACE2* (chrX_15596143), *IL-18* (chr11_112014152 and chr11_112020916), *IL-18R1* (chr2_102984684 and chr2_103010912), *IL-4R* (chr16_27358098), *IL-6* (chr7_22767433), *IL-7R* (chr5_35861152 and chr5_35876274), *TRAF6* (chr11_36518824), *IFNGR1* (chr6_137519780), *IL-12RB1* (chr19_18171886), *TEME50B* (chr21_34804966), *IL-13* (chr5_131995843), *NR112* (chr3_119526349), and *PTPN22* (chr1_114400590).

Similarly, we identified polymorphisms whose presence is potentially causative of RA. Among these, *IL-18R1* (chr2_102984624, chr2_102984671, and chr2_103013408), *IL-4R* (chr16_27366982), *IRF5* (chr7_128587351), *IL-12RB1* (chr19_18170384 and chr19_18173179), and *NR1I2* (chr3_119501506). Regarding polymorphisms with a causative trend but lacking statistical significance, we reported the polymorphism in IL-4R (chr16_27370209 and chr16_27375070) and *IRF5* (chr7_128586057 and chr7_128587724) genes. Meanwhile, among those that are not statistically significant but exhibit a protective trend, we identified the polymorphism in *IRF5* (chr7_128586057) and *ATG7* (chr3_11596348) genes.

Regarding SLE, we have pointed out several statistically significant polymorphisms in 11 genes (Table 5 and Table 6). Those with a potential protective role include *PARK2* (chr6_161990516), *IL-18* (chr11_112020916), *IL18R1* (chr2_103010912), *TMPRSS2* (chr21_42842854), *TRAF6* (chr11_36518824), *IL-12RB2* (chr19_18180367), and *PTPN22* (chr1_114414021). Whereas the polymorphisms with a potential causative effect comprise *PARK2* (chr6_161771219), *IRF5* (chr7_128586760), *MAPT* (chr17_44051927), *IL-12RB1* (chr19_18170773), and *SLC11A1* (chr2_219256076 and chr2_219259270). No statistically significant difference was identified for the polymorphisms in the genes *PARK2* (chr6_162394341), *IL-12RB1* (chr19_18180367), and *IL-12RB2* (chr1_67833501).

The polymorphisms identified in *ACE2* (chrX_15596143), *IL-18* (chr11_112014152), *IL-18R1* (chr2_103013408), *IFNGR1* (chr6_137519588), and *IL-2* (chr4_123377482) genes can be considered predisposing factors for the onset of systemic sclerosis. No statistically significant polymorphisms were identified that could be considered protective in the pathological context (Table 7 and Table 8).

In contrast, in SS disease, we identified several polymorphisms that can be considered protective within the pathological context (Table 9 and Table 10). The genes involved are *PARK2* (chr6_161781225 and chr6_161990516), *IL-18* (chr11_112020916), *IL-18R1* (chr2_102984684 and chr2_103010912), *IL-6* (chr7_22767433), *TMPRSS2* (chr21_42866107), *IFNGR1* (chr6_137519780), and *IL-12RB2* (chr1_67852335). Also, the polymorphisms identified in the genes *VDR* (chr12_48272895), *TMEM50B* (chr21_34804966), and *IL-2RA* (chr10_6061730) appear to have a role that tends towards protection in the pathological context, although the results are not statistically significant. Regarding the polymorphisms associated with the risk of disease onset, the interest genes are *IL-12A* (chr3_159713467), *IL18R1* (chr2_103013408 and chr2_103013388), *IL-4R* (chr16_27373966), *MAPT* (chr17_44101591), *IL-12RB1* (chr19_18191621), and *IFNGR2* (chr21_34805196). The same trend, albeit not statistically significant, was observed for the polymorphisms in the genes *VDR* (chr12_48238837, chr12_48250921, and chr12_48259057).

Additionally, we investigated whether mutations at specific sites we identified had also been observed in other conditions and whether they had been classified as benign or pathogenic. For this analysis, we utilized the ClinVar database (https://www.ncbi.nlm.nih.gov/clinvar/, accessed on 5 January 2024), which offers a comprehensive overview of various polymorphisms currently identified, primarily classifying them into pathogenic variants, benign variants, likely benign variants and variants of unknown significance (Table 11).

For most polymorphisms we identified, there was congruence with data in the ClinVar database, while for others, the benign or pathogenic effect was not corroborated by our findings. Specifically, for the polymorphism in the *IL-7R* gene (in the chr5_35871190 position), the literature reports a benign clinical significance, yet our analysis suggests a potential predisposing role in SRD conditions. Regarding the *VDR* gene (at the chr12_48272821 site), the polymorphism we identified appears to have a non-significant protective role, although it is categorized as likely pathogenic in the literature. In the case of polymorphisms identified at chr1_67833501 (*IL-12RB2*) and chr6_137519588 (*IFNGR1*), the benign significance reported in the literature was not confirmed by our results, which instead suggests these polymorphisms as predisposing in SLE and SSc, respectively. Similarly, in the context of SS, the *VDR* (chr12_48238837) and *IFNGR2* (chr21_34805196) genes, reported as benign in literature, were found in our analyses to be predisposing factors in the pathological context (Table 11).

## 3. Discussion

Genetic polymorphisms play a pivotal role in elucidating the pathogenic mechanisms underlying SRDs. Our investigative efforts have pinpointed several critical genes, each harboring unique polymorphisms that potentially contribute to the disease’s manifestations. In the arising discussion, we will explore these polymorphisms and examine their potential implications in the pathophysiology of SRDs. The immune system’s function is critically integral in the context of autoimmune diseases, underscoring the significance of identifying polymorphisms within pivotal genes that may exert either a beneficial or detrimental impact. Although many polymorphisms identified in this study lack empirical validation for their protective or predisposing effects, largely due to their novel association with SRDs, our findings suggest that mutations in adjacent but distinct chromosomal regions can lead to varied outcomes. Additionally, the consistent recurrence of specific mutations in some diseases hints at a uniform role in either protection or predisposition.

Several studies highlight the role of cytokines in the pathophysiological mechanisms underlying SRDs [29,30,31]. Cytokine anomalies, indicative of inflammation but not causative, serve as biomarkers and active players in disorders, with their diverse network influencing the balance between proinflammatory and anti-inflammatory responses.

Our investigation originated with IL-12A and IL-12RB2, driven by their crucial roles in immune response modulation. IL-12 is involved in the pathogenesis of immune rheumatic diseases, such as SLE and SS [32]. *IL-12A* encodes the p35 subunit of IL-12, a pivotal cytokine in initiating and sustaining Th1 immune responses [33,34]. *IL-12RB2* codes the beta 2 subunit of the IL-12 receptor, an essential element for IL-12 signaling. This particular receptor subunit is crucial for the effective response of both T cells and NK cells to IL-12 stimulation [35]. In the field of rheumatic disease, it has been shown that even small amounts of IL-12 can induce the production of proinflammatory cytokines, including IL-1 and TNF-α. Furthermore, these cytokines have been shown to play a significant role in contributing to the clinical manifestations observed in RA [36]. In our investigation, we detected distinctive polymorphic variations in the *IL-12A* and *IL-12RB2* genes with divergent implications. The *IL-12A* polymorphism at chr3_159713467 appears to be a significant predisposing factor for SS, while it appears to be predisposing but not statistically significant for SSc and SRDs, possibly enhancing Th1 responses [37,38]. In contrast, *IL-12RB2* polymorphism shows a protective effect for SRDs, including SLE and SS, potentially regulating IL-12 receptor function and moderating immune responses, thus possibly reducing the severity or prevalence of SRDs.

Our study extends to key cytokines, including IL-18 and its receptor IL-18R1. IL-18, a proinflammatory cytokine, and IL-18R1 are crucial in autoimmune rheumatic diseases and inflammasome pathways [39,40]. IL-18 triggers inflammatory responses upon its interaction with IL-18R1. Notably, a polymorphism in *IL-18* at chr11_112020916 offers protection against RA, SLE, and SS, likely by modulating IL-18 activity. Another *IL-18* variant, in the chr11_112014152 site, predisposes to diseases, possibly by enhancing inflammation. In *IL-18R1*, the chr2_103010912 polymorphism shows a protective effect in RA, SLE, and SS patients, indicating that changes in IL-18 signaling influence disease risk and progression. Conversely, the chr2_103013408 polymorphism in *IL-18R1* in SSc patients suggests an increased risk, underscoring the gene’s varied impact on disease pathways.

Following our analysis of *IL-18* polymorphism, we explored polymorphisms at the level of *IL-13*, *IL-6*, and other key genes. While IL-13’s exact role in rheumatic diseases is uncertain, it may influence their pathogenesis, especially in the production of autoantibodies such as rheumatoid factor (RF) [41]. In our analysis, a protective polymorphism was identified in the *IL-13* gene at chr5_131995843 in RA patients. IFNG, IL-17A, and IL-6 also play crucial roles in cytokine signaling, thereby influencing inflammatory responses. IL-6 and IFN-α/β are involved in the initiation of synovitis, specifically in synovial inflammation. IL-4, IL-18, IL-7, and IL-12 represent effector cell function and are involved in the perpetuation of synovitis and chronic synovitis [42]. In this context, we were particularly intrigued by the systemic effects of IL-6 and its extensive role in immunity and inflammation. IL-6 is multifunctional and central to regulating immune and inflammatory responses, linked to various autoimmune diseases, including AR, SS, SSc, and SLE [43]. It notably affects vascular tissues by enhancing AT1R expression, leading to endothelial dysfunction and inflammation [44]. IL-6 is key in the pathogenesis of inflammatory and autoimmune conditions, influencing diverse immune cells and pathways [45]. A protective polymorphism common in SRDs, AR, and SS, found at chr7_227674333, may modulate IL-6 production or activity, potentially leading to a more regulated immune response.

Our investigation then extended to IFNGR1, IFNGR2, and IFNG, which are crucial for mediating immune responses via IFN-γ. *IFNGR1* and *IFNGR2* encode the IFN-γ receptor’s subunits. It is critical for innate and adaptive immunity, is encoded by *IFNG*, and is linked to various autoimmune diseases [46]. Our findings show several polymorphisms in these genes affecting SRDs. In *IFNGR1*, a protective polymorphism at chr6_137519780, identified in SRDs, AR, and SS, may modulate IFN-γ signaling, probably leading to a less inflammatory response. Conversely, a predisposing polymorphism in *IFNGR1* at chr6_137519588, associated with SSc, might enhance IFN-γ receptor signaling, exacerbating immune response. In *IFNGR2*, the polymorphism at chr21_34805196 in SS suggests a predisposing effect, potentially affecting receptor functionality. Additionally, the *IFNG* polymorphism at chr12_68551941, protective but not statistically significant in SRDs, indicates a subtle influence on IFN-γ activity across SRDs, impacting the general immune response without directly affecting individual diseases.

Beyond the cytokine field, the investigation of how immune responses interact with blood pressure regulation emerges as a crucial area of research in SRDs. In fact, in the variety of mechanisms associated with RA, the renin–angiotensin system (RAS) plays a significant role in involving both ACE and ACE2 [47,48]. The balance between their respective activities is critical in regulating angiogenesis, a process central to the facilitation of inflammatory cell invasion. This, in turn, contributes to the hallmark structural damage observed in the articular tissues [49,50]. Additionally, osteopenia represents a significant factor in the pathogenesis of RA, given that progressive osteopenic degradation of affected joints leads to deformity and dysfunction [51]. Elevated ACE levels in RA synovial tissues, linked to increased stromal cell density [52], play a role in inflammation through the inactivation of pro-inflammatory agents like bradykinin and substance P [53]. The use of ACE inhibitors, like captopril, has shown symptom improvement in RA, indicating ACE’s role in disease pathogenesis [54,55,56,57,58]. A specific polymorphism in *ACE*, located at chr17_61566031, has been associated with RA. However, broader analysis revealed three *ACE* gene polymorphisms (chr17_61560763, chr17_61562063, and chr17_61564533) suggesting protective effects in SRDs, as detailed in Table 11. This finding underscores the genetic diversity of rheumatic diseases, where polymorphisms may have minor effects individually but become significant when synergized in the disease context, indicating potential overlapping pathogenic mechanisms.

Delving into the functions of PARK2 and PAR2, we recognized their diverging roles from traditional immunological pathways. While PARK2 is essential for mitochondrial function and cellular turnover, PAR2’s involvement in inflammation and rheumatic disease pathogenesis prompted our investigation. The *PARK2* gene, wich encodes the Parkin protein, is notably relevant in the context of neurogenetics, especially concerning Parkinson’s disease. It regulates mitochondrial function and the ubiquitin-proteasome system, which is essential for cellular balance. Parkin aids in degrading depolarized mitochondria through autophagy and interacts with the anti-apoptotic protein Bcl-2 by facilitating its mono-ubiquitination, thereby impacting Bcl-2 levels [59]. Recent research suggests a substantial role of autophagy and apoptosis in promoting self-tolerance and in the underlying regulatory mechanisms that influence susceptibility to, and immune regulation within, rheumatic diseases [60]. In RA, chronic synovitis, and systemic inflammation, marked by autoantibodies like RF and anti-citrullinated protein antibodies (ACPAs), lead to macrophage activation, contributing to synovitis and bone damage. Autophagy is crucial in presenting citrullinated peptides and ACPA production, with RA fibroblast-like synoviocytes showing high levels of citrullinated proteins. Abnormal autophagy in RA is associated with synovial hyperplasia and bone erosion, aiding the survival of these synoviocytes and osteoclastogenesis [61,62,63,64]. Genome-wide studies in SLE patients have identified SNPs in autophagy-related genes like *ATG5* and *ATG7* linked to increased disease risk. Genetic variations near *ATG5* correlate with the onset, progression, and specific clinical manifestations of SLE. Autophagy in SLE affects various immune responses, including monocyte-mediated clearance of apoptotic cells, regulation of type I interferon, and B- and T-cell functionality. It also contributes to cytokine production and heightened activation in T cells [65]. In SS, recent studies indicate a significant role of autophagy, particularly its dysregulation in T and B lymphocytes correlating with disease severity and activity. Autophagy results in upregulation in these cells and notably in salivary gland epithelial cells (SGECs), affecting cell survival and inflammatory responses. Excessive autophagy in SGECs, triggered by inflammation, aggravates SS by promoting abnormal cell activation and autoantigen redistribution [66].

In our analysis, *PARK2* carries a protective polymorphism (in the chr6_161990516 site) against SRDs, AR, SLE, and SS, suggesting a role in mitigating autoimmune responses. This protective effect could be attributed to PARK2’s involvement in cellular processes like autophagy and mitophagy, potentially influencing immune cell function and reducing inflammatory processes. Conversely, polymorphism in the chr6_161771219 site, identified as predisposing in SLE, indicates a potential alteration in these protective mechanisms.

Regarding PAR2, it plays a critical role in immune processes. This receptor significantly modulates inflammation via pro-inflammatory cytokines release that are involved in synovitis and adaptive antibody responses. The elevated expression of PAR2 in RA patient synovial cells, its contribution to IL-1β secretion in synovial fluid, and its role in bone remodeling highlight its significance in RA pathogenesis and its potential as a therapeutic target [67,68]. Our findings have highlighted two particularly intriguing polymorphisms concerning their contrasting roles. The polymorphism at chr3_119501596 is associated with protection, and the polymorphism located in chr3_119526349 shows a predisposing effect, highlighting the dual role of this specific gene.

We then focused on the distinct roles of PTPN22, SLC11A1, and IRF5 in immune regulation and autoimmunity. The *PTPN22* gene, crucial in T-cell receptor signaling, is notably associated with increased risk for rheumatic diseases like RA, SLE, and juvenile idiopathic arthritis (JIA). The R620W SNP variant in *PTPN22*, through altered phosphatase activity, disrupts T-cell activation, contributing to autoimmunity and the progression of these diseases [69]. The two polymorphisms we have identified are located at the site chr1_114400590 in RA and at the chr1_1144144021 site in SLE, and both exhibit protective effects. Notably, no polymorphisms with risk effects have emerged for any of the SRDs studied. The *SLC11A1* gene, key in immune regulation and macrophage function, is linked to the susceptibility and severity of rheumatic diseases such as RA and SLE [27,70]. Its polymorphisms affect macrophage response, including cytokine production, contributing to the pathogenesis of these conditions [71]. In our analysis, this gene presents two polymorphisms, both with a hypothetical predisposing significance in SLE, located at the chr2_219256076 and chr2_219259270 sites. The *IRF5* gene, important in immune regulation, is linked to rheumatic diseases like SLE, RA, and SS due to its influence on type I interferons and pro-inflammatory cytokines [72,73]. Our study has pinpointed specific polymorphisms in the *IRF5* gene affecting SRDs at chr7_128587351. It is identified as a risk factor for AR, possibly enhancing IRF5’s role in pro-inflammatory pathways and contributing to AR’s pathogenesis through abnormal inflammatory responses. Additionally, a polymorphism at chr7_128586760 is associated with an increased susceptibility to SLE, indicating that alterations in IRF5 could lead to dysregulated immune responses, including the overactivation of type I interferon pathways, central to SLE’s pathology.

Finally, our study encompassed TRAF6, MAPT, and PTPN22. TRAF6, integral to immune signaling and bone homeostasis, influences the NF-κB and MAPK pathways, impacting inflammation and osteoclast differentiation [74]. Dysregulation of TRAF6 is linked to rheumatic diseases such as RA, contributing to chronic inflammation, cytokine production, and joint destruction due to overactive TRAF6 pathways and increased bone erosion [75]. Our study found a protective polymorphism in the *TRAF6* gene at chr11_36518824, which may reduce overactive immune responses in SRDs, AR, and SLE. This variation potentially modulates inflammation and immune activation pathways, suggesting TRAF6’s role in maintaining immune balance and influencing the progression of these autoimmune conditions.

Our ivestigation of the MAPT gene aimed to explore the connections between neurology and immunology. Known for encoding the tau protein, traditionally associated with neurodegenerative disorders, recent studies have begun investigating MAPT’s role in autoimmune diseases [76,77]. Our research has identified two polymorphisms within the *MAPT* gene with predisposing effects in different SRDs. Polymorphism in chr17_44051927 has been found to predispose individuals to SLE, and its presence may impact the structure or function of the tau protein, influencing cellular and immune regulation processes. Altered tau protein could lead to immune response dysregulation or increased autoimmunity. Additionally, the chr17_44101591 polymorphism is associated with a higher risk for SS, suggesting MAPT’s involvement in SS through potential effects on cellular stability and immune functions. We may speculate that the alterations in tau protein could be associated with the principal symptoms of SS, such as glandular dysfunction and chronic inflammation. PTPN22 is integral in the modulation of immune responses, with a known role in the regulation of signaling in immune cells, specifically in T cells and B cells. It has garnered significant research interest in the field of autoimmune diseases, owing to its influence on immune tolerance and activation processes [78]. Our research has identified two polymorphisms, both exhibiting protective effects but in different rheumatic conditions. Polymorphism in chr1_114400590 shows a protective role in RA, and it may influence the phosphatase activity of PTPN22, leading to a modulation of T-cell and B-cell responses. Such an alteration could result in a reduced propensity for the autoimmunity that characterizes RA, possibly by enhancing immune tolerance or reducing aberrant immune activation. Similarly, polymorphism in chr1_114414021 has been found to exert a protective effect in SLE. The presence of this polymorphism might lead to changes in immune signaling pathways that are less conducive to the development of SLE, possibly by regulating lymphocyte activation and maintaining immune homeostasis more effectively.

The investigation of polymorphisms within rheumatic diseases presented in our study provides insight into their potential implications in a pathological context. Although our research lacks experimental validation of the underlying molecular mechanisms, it may provide a rationale for the investigation of various disease processes. Another limitation of our study is the lack of patients’ clinical data. While our investigation is based on the identification of specific SNPs, we acknowledge that correlation with clinical data can provide more exhaustive information regarding the implication of polymorphism in the disease context. In our plans, besides clinical data collection, we aim to include a broader spectrum of populations to improve our understanding of genetic factors in different ethnicities, considering the critical role of genetic diversity. 

## 4. Materials and Methods

### 4.1. Sample Collection and PBMCs Isolation

Our study involved the recruitment of two cohorts in the period between 2017 and 2019. We enrolled a total of 100 healthy control subjects (HCs) and 88 patients with SRDs, further categorized as 26 individuals diagnosed with RA, 21 with SS, 29 SSc patients, and 12 subjects with SLE. The diagnosis was established by qualified rheumatologists at the Rheumatology Unit of the University Hospital of Sassari according to international criteria.

Blood samples were collected from all individuals diagnosed with SRDs who were enrolled at the Rheumatology Unit of the University Hospital of Sassari, ensuring the exclusion of patients with any significant health conditions in addition to SRDs. In the selection of healthy control subjects recruited through the Transfusion Center at AOU, Sassari, Italy, careful matching was performed based on age and sex to the SRD cohort, aiming to substantially reduce the impact of these demographic variables on the analysis between the two groups. Before their inclusion in the study, all participants furnished written informed consent. The investigation adhered to the ethical principles outlined in the Declaration of Helsinki, and the study protocol received approval from the Institutional Review Board of the University of Cagliari (PG 2018/5643).

Peripheral whole blood samples were collected from both cohorts using K^+^-EDTA test tubes. Subsequently, Ficoll–Histopaque gradient centrifugation (Sigma-Aldrich, St. Louis, MO, USA) was employed to isolate peripheral blood mononuclear cells (PBMCs) from other blood components. PBMCs were then preserved at −80 °C in a solution of fetal bovine serum (FBS) and 10% dimethyl sulfoxide (DMSO) (Sigma-Aldrich, St. Louis, MO, USA) until use.

### 4.2. Genomic DNA Extraction

PBMCs were washed with Phosphate Buffer Saline 1X (PBS) and subsequently resuspended in 200 μL of PBS 1X. Genomic DNA extraction was carried out following the manufacturer’s instructions using the DNeasy Blood and Tissue Kit (Qiagen, Germantown, MD, USA). The DNA yield was determined using the Nanodrop One spectrophotometer (Thermo Scientific, Waltham, MA, USA), and DNA quality was assessed by measuring absorbance ratios at A260/A280 and A260/A230.

### 4.3. Library Preparation and Genomic DNA Sequencing

A panel of thirty genes associated with both innate and adaptive immune responses, as well as genes linked to SRDs SRDs, were sequenced. The library preparation and DNA sequencing procedures were conducted at the CRS4 Research Center located in Pula, Sardegna, Italy. For gene panel analysis, two AmplySeq primer pools were designed and purchased from Illumina. The PCR protocol and reagents were adapted from the QIAGEN Application Note (Qiagen 1104745, 10/2016) to amplify the entire length of each gene. Quantification of PCR products was carried out using Qubit, and equimolar amounts were pooled. Gene-panel PCR pools were grouped to achieve the desired sequencing coverage. Libraries were constructed using Nextera DNA Flex with 100 ng of DNA and indexed using the IDT for the Illumina Nextera DNA UD Indexes Primer Set (Illumina, San Diego, CA, USA). Following PCR, the products were purified with 1X AMPure XP beads (Beckman Coulter, Brea, CA, USA), and library quantification was performed using Qubit. The loading pool, comprising 188 samples (88 from SRDs and 100 from HCs subjects), was diluted to 9 pM before sequencing using the MiSeq Reagent Kit v3 600-cycle (Illumina). Demultiplexing and fastq file generation were carried out using the BaseSpace Sequence Hub (Illumina), and each sample underwent two distinct analyses. Gene panel sequences were subjected to analysis through an in-house pipeline.

### 4.4. Biostatistical Analyses

The analyses conducted allowed the identification of polymorphisms that are statistically associated with the onset of the SRDS. The chosen level of statistical significance was set at Pr(>|t|) < 0.05, used to assess the significance of predictors in regression models. This notation indicates the *p*-value associated with the *t*-test, where a lower value suggests stronger evidence against the null hypothesis, typically leading to the conclusion that the observed effect is statistically significant. The identification has been performed by using a multivariate regression carried out with the generalized linear mixed models (GLMMs) implemented in the R-package lme4 [79] within the R environment (version 4.1.1; R Core Team, https://www.r-project.org/, accessed on 1 January 2024). The regression with GLMM has also been applied to examine the relationship between the SRDs and nucleotide substitution at given loci. The multivariate regression analysis was conducted using a backward selection procedure where non-significant covariates were systematically removed step by step until the final model was attained. The final model, comprising only statistically significant covariates that can serve as predictors, was utilized to calculate the odds of developing rheumatic diseases. The probability has been quantified using the odds ratio parameter (OR) with a 97.5% confidence interval, calculated using the R package “oddsratio” (available at https://cran.r-project.org/web/packages/oddsratio/ accessed on 1 January 2024). Analyses were conducted individually on each gene for SRDs and separately for each specific illness.

## 5. Conclusions

The presence of specific polymorphisms in the disease context, which may influence disease severity or confer protection, plays a key role in determining clinical outcomes. Therefore, the collective impact of these polymorphisms, rather than their individual effects, may ultimately dictate the course of disease progression and the patient’s prognosis.

## Figures and Tables

**Table 1:** Results of multivariate analysis conducted individually on each gene for the SRDs. The table shows the variables (potential predictors) with statistical significance in terms of the occurrence of the illnesses and the summary of found coefficients. Significance codes: Pr(>|t|) < 0.001 = ‘***’, <0.01 = ‘**’, <0.05 = ‘*’, >0.05 = Non-significant code ‘.’.

Gene	Position	Mutation	Estimate	Std. Error	t Value	Pr(>|t|)
*ACE*	chr17_61560763	T → C rs4311 intronic	−0.18308	0.08180	−2.238	0.0272 *
	chr17_61562063	G → C rs12720722 intronic	−0.86364	0.43233	−1.998	0.0481 *
	chr17_61564533	C → G rs55907121 intronic	−0.86364	0.43233	−1.998	0.0481 *
*IL-12A*	chr3_159713467	G → A rs568408 3′UTR	0.28182	0.16042	1.757	0.0816.
*PARK2*	chr6_161781225	C → T rs1801334 missense	−0.39006	0.19423	−2.008	0.0470 *
	chr6_161990516	G → C rs3765475 intronic	−0.20270	0.07892	−2.568	0.0115 *
	chr6_162394341	C → T rs146173584 missense	−0.87114	0.42823	−2.034	0.0442 *
*LAG3*	chr12_6883722	T → C rs201769189 intronic	−0.74359	0.44040	−1.688	0.094.
*ACE2*	chrX_15589725	C → G rs4646168 intronic	−0.76316	0.42701	−1.787	0.0766.
	chrX_15590120	T → C rs2301692 intronic	−0.76316	0.30325	−2.517	0.0132 *
	chrX_15613148	T → C - intronic	−0.76316	0.42701	−1.787	0.0766.
*IFNG*	chr12_68551941	A → G - intronic	−0.74359	0.44040	−1.688	0.094.
*IL-17A*	chr6_52053771	C → T rs17881747 intronic	−0.74359	0.44040	−1.688	0.094.
*IL-18*	chr11_112020916	T → G rs549908 synonymous	−0.24638	0.08060	−3.057	0.00278 **
*IL-18R1*	chr2_103010912	C → T rs1420096 intronic	−0.32080	0.08225	−3.900	0.000162 ***
	chr2_103013408	C → T rs7570821 3′UTR	0.21779	0.08666	2.513	0.013349 *
*IL-4R*	chr16_27353479	C → T rs17548704 synonymous	−0.35221	0.20020	−1.759	0.0812.
*IL-5*	chr5_131878992	A → C - intronic	−0.74359	0.44040	−1.688	0.094.
*IL-6*	chr7_22767433	A → G rs2069832 intronic	−0.18301	0.08813	−2.077	0.0401 *
	chr7_22768249	G → T rs2066992 intronic	−0.25826	0.15166	−1.703	0.0913.
*IL-7R*	chr5_35857235	C → G rs1494561 intronic	−0.39327	0.09232	−4.260	4.22 × 10^−5^ ***
	chr5_35861159	A → G rs969128 intronic	−0.18606	0.08934	−2.083	0.0395 *
	chr5_35871190	G → A rs1494555 missense	0.21247	0.09005	2.359	0.0200 *
*IRF5*	chr7_128586057	C → T rs139688977 synonymous	−0.75652	0.24991	−3.027	0.00304 **
*MAPT*	chr17_44060845	T → C - synonymous	−0.86111	0.43926	−1.960	0.0524.
	chr17_44101591	GA → G, GAA - 3′UTR	0.16975	0.08679	1.956	0.0529.
*TMPRSS2*	chr21_42866107	T → C rs2838042 intronic	−0.27348	0.09034	−3.027	0.00304 **
*TRAF6*	chr11_36518824	T → TA rs3830511 splicing	−0.22306	0.08279	−2.694	0.00811 **
*VDR*	chr12_48272821	C → A - missense	−0.74359	0.44040	−1.688	0.094.
*IFNGR1*	chr6_137519780	T → C rs3799488 splicing	−0.28000	0.11064	−2.531	0.0127 *
*IL-12RB1*	chr19_18184462	G → A - intronic	−0.74359	0.44040	−1.688	0.094.
*IL-12RB2*	chr1_67833867	C → T rs145750129 intronic	−0.85714	0.43712	−1.961	0.0523.
	chr1_67852335	G → A rs2228420 synonymous	−0.17714	0.08326	−2.128	0.0355 *

**Table 2:** Results of odds ratios calculation from multivariate analysis applied to the statistically significant variables representing useful predictors (see Table 1) for the SRDs. Calculation was performed on the final model with 10% increment steps across the whole predictor distribution. ° Range of odds ratio within the 10 steps that split the predictor distribution.

Gene	Position	Odds Ratio	CI_Low (2.5%) °	CI_High (97.5%) °
*ACE*	chr17_61560763	0.833	0.709	0.978
	chr17_61562063	0.422	0.181	0.984
	chr17_61564533	0.422	0.181	0.984
*IL-12A*	chr3_159713467	1.326	0.968	1.815
*PARK2*	chr6_161781225	0.677	0.463	0.991
	chr6_161990516	0.817	0.700	0.953
	chr6_162394341	0.418	0.181	0.969
*LAG3*	chr12_6883722	0.475	0.201	1.127
*ACE2*	chrX_15589725	0.466	0.202	1.077
	chrX_15590120	0.466	0.257	0.845
	chrX_15613148	0.466	0.202	1.077
*IFNG*	chr12_68551941	0.475	0.201	1.127
*IL-17A*	chr6_52053771	0.475	0.201	1.127
*IL-18*	chr11_112020916	0.782	0.667	0.915
*IL-18R1*	chr2_103010912	0.726	0.618	0.852
	chr2_103013408	1.243	1.049	1.473
*IL-4R*	chr16_27353479	0.703	0.475	1.041
*IL-5*	chr5_131878992	0.475	0.201	1.127
*IL-6*	chr7_22767433	0.833	0.701	0.99
	chr7_22768249	0.772	0.574	1.04
*IL-7R*	chr5_35857235	0.675	0.563	0.809
	chr5_35861159	0.830	0.697	0.989
	chr5_35871190	1.237	1.037	1.475
*IRF5*	chr7_128586057	0.469	0.288	0.766
*MAPT*	chr17_44060845	0.423	0.179	1.000
	chr17_44101591	1.185	1.000	1.405
*TMPRSS2*	chr21_42866107	0.761	0.637	0.908
*TRAF6*	chr11_36518824	0.8	0.68	0.941
*VDR*	chr12_48272821	0.475	0.201	1.127
*IFNGR1*	chr6_137519780	0.756	0.608	0.939
*IL-12RB1*	chr19_18184462	0.475	0.201	1.127
*IL-12RB2*	chr1_67833867	0.424	0.180	1.000
	chr1_67852335	0.838	0.712	0.986

**Table 3:** Results of multivariate analysis conducted individually on each gene for RA. The table shows the variables (potential predictors) with statistical significance with the occurrence of the illness and the summary of found coefficients. Significance codes: Pr(>|t|) < 0.001 = ‘***’, <0.01 = ‘**’, <0.05 = ‘*’, >0.05 = Non-significant code ‘.’.

Gene	Position	Mutation	Estimate	Std. Error	t Value	Pr(>|t|)
*ACE*	chr17_61566031	G → A rs4343 synonymous	−0.34968	0.12749	−2.743	0.00825 **
*PARK2*	chr6_161990516	G → C rs3765475 intronic	−0.42529	0.12239	−3.475	0.00102 **
*TMEM50B*	chr21_34804966	T → C rs1532 3′UTR	−0.3948	0.1762	−2.241	0.0292 *
*ACE2*	chrX_15596143	C → CATAAG rs113691336 intronic	−0.30952	0.15045	−2.057	0.0445 *
*ATG7*	chr3_11596348	C → T rs14016 3′UTR	−0.30000	0.17185	−1.746	0.0866.
*IL-13*	chr5_131995843	T → C rs1295686 intronic	−0.6200	0.2018	−3.072	0.00332 **
*IL-18*	chr11_112014152	C → G rs360728 3′UTR	−0.29167	0.12811	−2.277	0.02686 *
	chr11_112020916	T → G rs549908 synonymous	−0.34375	0.12404	−2.771	0.00768 **
*IL-18R1*	chr2_102984624	C → G rs1362348 intronic	0.33233	0.14883	2.233	0.03006 *
	chr2_102984671	T → A rs1882348 intronic	0.39441	0.14562	2.709	0.00923 **
	chr2_102984684	G → A rs1558627 intronic	−0.54781	0.17369	−3.154	0.00272 **
	chr2_103010912	C → T rs1420096 intronic	−0.56936	0.16700	−3.409	0.00130 **
	chr2_103013408	C → T rs7570821 3′UTR	0.28678	0.12573	2.281	0.02685 *
*IL-4R*	chr16_27358098	A → C rs2301807 intronic	−0.7436	0.1533	−4.849	1.21 × 10^−5^ ***
	chr16_27366982	T → C rs3024636 intronic	0.4103	0.1818	2.256	0.0284 *
	chr16_27370209	A → G - intronic	0.7436	0.4199	1.771	0.0826.
	chr16_27375070	T → C rs3024679 synonymous	0.7436	0.4199	1.771	0.0826.
*IL-6*	chr7_22767433	A → G rs2069832 intronic	−0.4570	0.1333	−3.427	0.00117 **
*IL-7R*	chr5_35861152	C → G rs11567705 intronic	−0.4486	0.1307	−3.432	0.00117 **
	chr5_35876274	A → G rs3194051 missense	−0.4220	0.1253	−3.367	0.00142 **
*IRF5*	chr7_128586057	C → T rs139688977 synonymous	−0.5263	0.2776	−1.896	0.0635.
	chr7_128587351	CACTCTGCAGCCGCCCACTCTGCGGCCGCCT → C rs199508964; rs60344245 nonframeshift	0.3596	0.1533	2.347	0.0228 *
	chr7_128587724	C → T rs190039770 intronic	0.4737	0.2776	1.706	0.0939.
*NR1I2*	chr3_119501506	T → G rs3814056 intronic	0.7143	0.3349	2.133	0.03760 *
	chr3_119526349	G → A rs1464603 intronic	−0.3985	0.1313	−3.036	0.00372 **
*PTPN22*	chr1_114400590	C → T rs55730164 intronic	−0.49020	0.22767	−2.153	0.0358 *
*TRAF6*	chr11_36518824	T → TA rs3830511 splicing	−0.5048	0.1217	−4.148	0.000119 ***
*IFNGR1*	chr6_137519780	T → C rs3799488 splicing	−0.42174	0.16705	−2.525	0.0146 *
*IL-12RB1*	chr19_18170384	G → A rs3746190 3′UTR	0.4263	0.1794	2.376	0.02121 *
	chr19_18171886	A → G rs383483 intronic	−0.5586	0.1827	−3.057	0.00352 **
	chr19_18173179	C → T - intronic	1.0226	0.5000	2.045	0.04592 *

**Table 4:** Results of odds ratios calculation from multivariate analysis applied to the statistically significant variables representing useful predictors for RA (see Table 3). Calculation was performed on the final model with 10% increment steps across the whole predictor distribution. ° Range of odds ratio within the 10 steps that split the predictor distribution.

Gene	Position	Odds Ratio	CI_Low (2.5%) °	CI_High (97.5%) °
*ACE*	chr17_61566031	0.705	0.549	0.905
*PARK2*	chr6_161990516	0.654	0.514	0.831
*TMEM50B*	chr21_34804966	0.674	0.477	0.952
*ACE2*	chrX_15596143	0.734	0.546	0.985
*ATG7*	chr3_11596348	0.741	0.529	1.038
*IL-13*	chr5_131995843	0.538	0.362	0.799
*IL-18*	chr11_112014152	0.747	0.581	0.960
	chr11_112020916	0.709	0.556	0.904
*IL-18R1*	chr2_102984624	1.394	1.041	1.866
	chr2_102984671	1.484	1.115	1.974
	chr2_102984684	0.578	0.411	0.813
	chr2_103010912	0.566	0.408	0.785
	chr2_103013408	1.332	1.041	1.704
*IL-4R*	chr16_27358098	0.475	0.352	0.642
	chr16_27366982	1.507	1.055	2.153
	chr16_27370209	2.103	0.924	4.791
	chr16_27375070	2.103	0.924	4.791
*IL-6*	chr7_22767433	0.633	0.488	0.822
*IL-7R*	chr5_35861152	0.639	0.494	0.825
	chr5_35876274	0.656	0.513	0.838
*IRF5*	chr7_128586057	0.591	0.343	1.01
	chr7_128587351	1.433	1.061	1.935
	chr7_128587724	1.606	0.932	2.767
*NR1I2*	chr3_119501506	2.043	1.060	3.938
	chr3_119526349	0.671	0.519	0.868
*PTPN22*	chr1_114400590	0.613	0.392	0.957
*TRAF6*	chr11_36518824	0.604	0.476	0.766
*IFNGR1*	chr6_137519780	0.656	0.473	0.91
*IL-12RB1*	chr19_18170384	1.532	1.078	2.177
	chr19_18171886	0.572	0.400	0.818
	chr19_18173179	2.780	1.044	7.408

**Table 5:** Results of multivariate analysis conducted individually on each gene for SLE. The table shows the variables (potential predictors) with statistical significance with the occurrence of the illness and the summary of found coefficients. Significance codes: Pr(>|t|) < 0.001 = ‘***’, <0.01 = ‘**’, <0.05 = ‘*’, >0.05 = Non-significant code ‘.’.

Gene	Position	Mutation	Estimate	Std. Error	t Value	Pr(>|t|)
*PARK2*	chr6_161771219	G → A rs149953814 missense	0.5417	0.2599	2.084	0.043746 *
	chr6_161990516	G → C rs3765475 intronic	−0.3417	0.1397	−2.446	0.019066 *
	chr6_162394341	C → T rs146173584 missense	−1.0083	0.5096	−1.979	0.054942.
*IL-18*	chr11_112020916	T → G rs549908 synonymous	−0.29221	0.13250	−2.205	0.0331 *
*IL-18R1*	chr2_103010912	C → T rs1420096 intronic	−0.5376	0.1317	−4.083	0.000201 ***
*IRF5*	chr7_128586760	C → T rs73461593 3′UTR	0.75610	0.31097	2.431	0.019498 *
*MAPT*	chr17_44051927	T → C rs148721531 5′UTR	0.75610	0.31097	2.431	0.019498 *
*PTPN22*	chr1_114414021	CTT → C, CT, CTTT - intronic	−0.4821	0.1327	−3.634	0.00077 ***
*SLC11A1*	chr2_219256076	T → G rs368624123 intronic	1.000	4.399 × 10^−1^	2.273	0.0285 *
	chr2_219259270	G → A rs2279015 intronic	3.548 × 10^−1^	1.478 × 10^−1^	2.400	0.0211 *
*TMPRSS2*	chr21_42842854	C → A rs465576 intronic	−0.8611	0.1339	−6.433	1.05 × 10^−7^ ***
*TRAF6*	chr11_36518824	T → TA rs3830511 splicing	−0.3580	0.1505	−2.378	0.02213 *
*IL-12RB1*	chr19_18170773	A → G rs199686420 synonymous	0.61081	0.19171	3.186	0.0028 **
	chr19_18180367	G → A rs748284308 missense	0.81081	0.40776	1.988	0.0536.
*IL-12RB2*	chr1_67833501	G → A rs2252596 splicing	0.4232	0.2443	1.732	0.09093.
	chr1_67852335	G → A rs2228420 synonymous	−0.3925	0.1306	−3.006	0.00456 **

**Table 6:** Results of odds ratios calculation from multivariate analysis applied to the statistically significant variables representing useful predictors for SLE (see Table 5). Calculation was performed on the final model with 10% increment steps across the whole predictor distribution. ° Range of odds ratio within the 10 steps that split the predictor distribution.

Gene	Position	Odds Ratio	CI_Low (2.5%) °	CI_High (97.5%) °
*PARK2*	chr6_161771219	1.719	1.033	2.861
	chr6_161990516	0.711	0.540	0.934
	chr6_162394341	0.365	0.134	0.990
*IL-18*	chr11_112020916	0.747	0.576	0.968
*IL-18R1*	chr2_103010912	0.584	0.451	0.756
*IRF5*	chr7_128586760	2.13	1.158	3.918
*MAPT*	chr17_44051927	2.13	1.158	3.918
*PTPN22*	chr1_114414021	0.618	0.476	0.801
*SLC11A1*	chr2_219256076	2.718	1.148	6.438
	chr2_219259270	1.426	1.067	1.905
*TMPRSS2*	chr21_42842854	0.423	0.325	0.549
*TRAF6*	chr11_36518824	0.699	0.521	0.939
*IL-12RB1*	chr19_18170773	1.842	1.265	2.682
	chr19_18180367	2.250	1.012	5.003
*IL-12RB2*	chr1_67833501	1.527	0.946	2.465
	chr1_67852335	0.675	0.523	0.872

**Table 7:** Results of multivariate analysis conducted individually on each gene for SSc. The table shows the variables (potential predictors) with statistical significance with the occurrence of the illness and the summary of found coefficients. Significance codes: Pr(>|t|) < 0.01 = ‘**’, <0.05 = ‘*’, >0.05 = Non-significant code ‘.’.

Gene	Position	Mutation	Estimate	Std. Error	t Value	Pr(>|t|)
*IL-12A*	chr3_159713467	G → A rs568408 3′UTR	0.5439	0.2925	1.859	0.068.
*ACE2*	chrX_15596143	C → CATAAG rs113691336 intronic	0.37054	0.12220	3.032	0.00363 **
*IL-18*	chr11_112014152	C → G rs360728 3′UTR	0.3992	0.1310	3.049	0.00346 **
*IL-18R1*	chr2_103013408	C → T rs7570821 3′UTR	0.29397	0.13567	2.167	0.0344 *
*IL-2*	chr4_123377482	C → A rs2069763 synonymous	0.2658	0.1266	2.100	0.040067 *
*IL-4R*	chr16_27373980	C → T rs1805013 missense	−1.00000	0.57080	−1.752	0.0852.
	chr16_27374927	T → G rs1805016 missense	0.53571	0.29295	1.829	0.0727.
*IRF5*	chr7_128586057	C → T rs139688977 synonymous	−0.54902	0.28870	−1.902	0.0623.
	chr7_128588475	C → A rs75282194 intronic	−0.38235	0.20974	−1.823	0.0735.
*NR1I2*	chr3_119501506	T → G rs3814056 intronic	0.5439	0.2925	1.859	0.068.
*IFNGR1*	chr6_137519588	A → C rs11914 synonymous	0.33714	0.12552	2.686	0.00942 **

**Table 8:** Results of odds ratios calculation from multivariate analysis applied to the statistically significant variables representing useful predictors SSc (see Table 7). Calculation was performed on the final model with 10% increment steps across the whole predictor distribution. ° Range of odds ratio within the 10 steps that split the predictor distribution.

Gene	Position	Odds Ratio	CI_Low (2.5%) °	CI_High (97.5%) °
*IL-12A*	chr3_159713467	1.723	0.971	3.056
*ACE2*	chrX_15596143	1.449	1.14	1.84
*IL-18*	chr11_112014152	1.491	1.153	1.927
*IL-18R1*	chr2_103013408	1.342	1.028	1.75
*IL-2*	chr4_123377482	1.305	1.018	1.672
*IL-4R*	chr16_27373980	0.368	0.120	1.126
	chr16_27374927	1.709	0.962	3.034
*IRF5*	chr7_128586057	0.578	0.328	1.017
	chr7_128588475	0.682	0.452	1.029
*NR1I2*	chr3_119501506	1.723	0.971	3.056
*IFNGR1*	chr6_137519588	1.401	1.095	1.792

**Table 9:** Results of multivariate analysis conducted individually on each gene for SS. The table shows the variables (potential predictors) with statistical significance with the occurrence of the illness and the summary of found coefficients. Significance codes: Pr(>|t|) < 0.001 = ‘***’, <0.01 = ‘**’, <0.05 = ‘*’, >0.05 = Non-significant code ‘.’.

Gene	Position	Mutation	Estimate	Std. Error	t Value	Pr(>|t|)
*IL-12A*	chr3_159713467	G → A rs568408 3′UTR	0.64583	0.24387	2.648	0.0108 *
*PARK2*	chr6_161781225	C → T rs1801334 missense	−0.52273	0.25695	−2.034	0.047349 *
	chr6_161990516	G → C rs3765475 intronic	−0.47727	0.11991	−3.980	0.000227 ***
*TMEM50B*	chr21_34804966	T → C rs1532 3′UTR	−0.3587	0.1969	−1.822	0.074420.
*IL-18*	chr11_112020916	T → G rs549908 synonymous	−0.46364	0.12421	−3.733	0.000485 ***
*IL-18R1*	chr2_102984684	G → A rs1558627 intronic	−0.37222	0.12595	−2.955	0.004869 **
	chr2_103010912	C → T rs1420096 intronic	−0.40153	0.13407	−2.995	0.004370 **
	chr2_103013388	TG → T rs11465656; rs397897634 3′UTR	0.96845	0.26910	3.599	0.000766 ***
	chr2_103013408	C → T rs7570821 3′UTR	0.30197	0.12154	2.485	0.016587 *
*IL-2RA*	chr10_6061730	TC → T rs28360486 intronic	−0.44681	0.22674	−1.971	0.0543.
*IL-4R*	chr16_27373966	C → T rs2234899 synonymous	0.64583	0.24387	2.648	0.0108 *
*IL-6*	chr7_22767433	A → G rs2069832 intronic	−0.4248	0.1444	−2.941	0.00495 **
*MAPT*	chr17_44101591	GA → G, GAA - 3′UTR	0.36134	0.13882	2.603	0.012132 *
*TMPRSS2*	chr21_42866107	T → C rs2838042 intronic	−0.33782	0.14000	−2.413	0.0195 *
*VDR*	chr12_48238837	C → A rs7975232 intronic	0.3147	0.1711	1.839	0.0722.
	chr12_48250921	T → G - missense	0.9306	0.4901	1.899	0.0637.
	chr12_48259057	T → C rs529794948 intronic	0.9306	0.4901	1.899	0.0637.
	chr12_48272895	A → G rs2228570 startloss	−0.3751	0.1895	−1.980	0.0536.
*IFNGR1*	chr6_137519780	T → C rs3799488 splicing	−0.3762	0.1678	−2.241	0.0295 *
*IFNGR2*	chr21_34805196	TC → T, * rs193922682 intronic	0.31944	0.14340	2.228	0.030433 *
*IL-12RB1*	chr19_18191621	G → T rs11673460 intronic	0.52381	0.18933	2.767	0.00792 **
*IL-12RB2*	chr1_67852335	G → A rs2228420 synonymous	−0.3589	0.1349	−2.661	0.0105 *

**Table 10:** Results of odds ratios calculation from multivariate analysis applied to the statistically significant variables representing useful predictors for SS (see Table 9). Calculation was performed on the final model with 10% increment steps across the whole predictor distribution. ° Range of odds ratio within the 10 steps that split the predictor distribution.

Gene	Position	Odds Ratio	CI_Low (2.5%) °	CI_High (97.5%) °
*IL-12A*	chr3_159713467	1.908	1.183	3.077
*PARK2*	chr6_161781225	0.593	0.358	0.981
	chr6_161990516	0.620	0.491	0.785
*TMEM50B*	chr21_34804966	0.699	0.475	1.028
*IL-18*	chr11_112020916	0.629	0.493	0.802
*IL-18R1*	chr2_102984684	0.689	0.538	0.882
	chr2_103010912	0.669	0.515	0.870
	chr2_103013388	2.634	1.554	4.463
	chr2_103013408	1.353	1.066	1.716
*IL-2RA*	chr10_6061730	0.64	0.41	0.998
*IL4R*	chr16_27373966	1.908	1.183	3.077
*IL-6*	chr7_22767433	0.654	0.493	0.868
*MAPT*	chr17_44101591	1.435	1.093	1.884
*TMPRSS2*	chr21_42866107	0.713	0.542	0.939
*VDR*	chr12_48238837	1.370	0.980	1.916
	chr12_48250921	2.536	0.970	6.627
	chr12_48259057	2.536	0.970	6.627
	chr12_48272895	0.687	0.474	0.996
*IFNGR1*	chr6_137519780	0.686	0.494	0.954
*IFNGR2*	chr21_34805196	1.376	1.039	1.823
*IL-12RB1*	chr19_18191621	1.688	1.165	2.447
*IL-12RB2*	chr1_67852335	0.698	0.536	0.91

**Table 11:** Comprehensive overview of identified polymorphisms, including their specific locations, that have shown statistical significance (Pr(>|t|) < 0.05) when comparing patients with control groups. The table details the pathology in which they are involved, specifying whether they are classified as reduced or increased risk according to our analysis. Additionally, the table presents existing scientific evidence (based on ClinVar database—PMID: 29165669, accessed on 5 January 2024) regarding the polymorphisms’ involvement in different diseases and their clinical significance. *Pathogenic variants* are mutations identified in DNA that are either established as disease-causing in prior studies or predicted to disrupt genetic functions. *Benign variants* represent innocuous DNA changes, frequently encountered in the general population (>1%) and not impacting health. *Likely benign variants* appear non-detrimental, resembling other non-problematic genetic alterations. *Variants of unknown significance* are anonymities in genetics, characterized by DNA changes with unclear implications due to inadequate data.

Gene Name	Position	Conditions	Risk		ClinVar (PMID: 29165669)	
			Reduced	Increased	Conditions	Clinical Significance
*ACE*	chr17_61560763	SRDs	✓			Benign
	chr17_61562063	SRDs	✓			
	chr17_61564533	SRDs	✓			
	chr17_61566031	AR	✓		Renotubular dysgenesis	Benign
*IL-12A*	chr3_159713467	SS		✓		
*PARK2*	chr6_161781225	SRDs, SS	✓		Autosomal recessive juvenile Parkinson’s disease 2. Lung cancer. Neoplasm of ovary.	Benign/likely benign
	chr6_161990516	SRDs, AR, SLE, SS	✓			Benign
	chr6_162394341	SRDs	✓		Autosomal recessive juvenile Parkinson’s disease 2	Uncertain significance
	chr6_161771219	SLE		✓	Autosomal recessive juvenile Parkinson’s disease 2	Conflicting interpretations of pathogenicity
*ACE2*	chrX_15590120	SRDs	✓			
	chrX_15596143	AR	✓			
		SLE		✓		
*IL-18*	chr11_112020916	SRDs, AR, SLE, SS	✓		Three vessel coronary disease	Benign
	chr11_112014152	AR	✓			
		SSc		✓		
*IL-18R1*	chr2_103010912	SRDs, AR, SLE, SS	✓			
	chr2_102984684	AR, SS	✓			
	chr2_102984624	AR		✓		
	chr2_103013408	SRDs, AR, SSc, SS		✓		
	chr2_102984671	AR		✓		
*IL-4R*	chr16_27358098	AR	✓			
	chr16_27366982	AR		✓		
	chr16_27373966	SS		✓		
*IL-6*	chr7_22767433	SRDs, AR, SS	✓			
*IL-7R*	chr5_35857235	SRDs	✓			Benign
	chr5_35861159	SRDs	✓			
	chr5_35861152	AR	✓			Benign
	chr5_35876274	AR	✓		Immunodeficiency 104	Benign
	chr5_35871190	SRDs		✓	Immunodeficiency 104	Likely benign
*IRF5*	chr7_128586057	SRDs	✓			Likely benign
	chr7_128587315	AR		✓		
	chr7_128586760	SLE		✓		
*MAPT*	chr17_44051927	SLE		✓		
	chr17_44101591	SS		✓		
*TMPRSS2*	chr21_42866107	SRDs, SS	✓			
	chr21_42842854	SLE	✓			
*TRAF6*	chr11_36518824	SRDs, AR, SLE	✓			Benign
*IFNGR1*	chr6_137519780	SRDs, AR, SS	✓		Disseminated atypical mycobacterial infection. Immunodeficiency 27A.	Benign
	chr6_137519588	SSc		✓		
*IL-12RB1*	chr19_18171886	AR	✓			
	chr19_18170384	AR		✓		
	chr19_18173179	AR		✓		
	chr19_18170773	SLE		✓	Mendelian susceptibility to mycobacterial diseases due to complete IL12RB1 deficiency	Conflicting interpretations of pathogenicity
	chr19_18191621	SS		✓		
*IL-12RB2*	chr1_67852335	SRDs, SLE, SS	✓			Benign
*TMEM50B*	chr21_34804966	AR	✓			
*IL-13*	chr5_131995843	AR	✓			
*NR1I2*	chr3_119526349	AR	✓			
	chr3_119501506	AR		✓		
*PTPN22*	chr1_114400590	AR	✓			
	chr1_114414021	SLE	✓			
*SLC11A1*	chr2_219256076	SLE		✓		
	chr2_219259270	SLE		✓		
*IL-2*	chr4_123377482	SSc		✓		
*IFNGR2*	chr21_34805196	SS		✓	Disseminated atypical mycobacterial infection. Immunodeficiency 27A.	Benign

## Data Availability

Data is contained within the article.
